# Percentage density, Wolfe's and Tabár's mammographic patterns: agreement and association with risk factors for breast cancer

**DOI:** 10.1186/bcr1308

**Published:** 2005-08-25

**Authors:** Inger T Gram, Yngve Bremnes, Giske Ursin, Gertraud Maskarinec, Nils Bjurstam, Eiliv Lund

**Affiliations:** 1 Institute of Community Medicine, University of Tromsø, Breivika, Norway; 2 Institute for Nutrition Research, University of Oslo, Norway; 3 Department of Preventive Medicine, University of Southern California, Keck School of Medicine, Los Angeles, CA, USA; 4 Cancer Research Center of Hawaii, University of Hawaii, Honolulu, HI, USA; 5 Department of Radiology, Center for Breast Imaging, University Hospital of North Norway, Tromsø, Norway

## Abstract

**Introduction:**

The purpose of this report was to classify mammograms according to four methods and to examine their agreement and their relationship to selected risk factors for breast cancer.

**Method:**

Mammograms and epidemiological data were collected from 987 women, aged 55 to 71 years, attending the Norwegian Breast Cancer Screening Program. Two readers each classified the mammograms according to a quantitative method (Cumulus or Madena software) and one reader according to two qualitative methods (Wolfe and Tabár patterns). Mammograms classified in the reader-specific upper quartile of percentage density, Wolfe's P2 and DY patterns, or Tabár's IV and V patterns, were categorized as high-risk density patterns and the remaining mammograms as low-risk density patterns. We calculated intra-reader and inter-reader agreement and estimated prevalence odds ratios of having high-risk mammographic density patterns according to selected risk factors for breast cancer.

**Results:**

The Pearson correlation coefficient was 0.86 for the two quantitative density measurements. There was moderate agreement between the Wolfe and Tabár classifications (Kappa = 0.51; 95% confidence interval 0.46 to 0.56). Age at screening, number of children and body mass index (BMI) showed a statistically significant inverse relationship with high-risk density patterns for all four methods (all *P *< 0.05). After adjustment for percentage density, the Wolfe classification was not associated with any of the risk factors for breast cancer, whereas the association with number of children and BMI remained statistically significant for the Tabár classification. Adjustment for Wolfe or Tabár patterns did not alter the associations between these risk factors and percentage mammographic density.

**Conclusion:**

The four assessments methods seem to capture the same overall associations with risk factors for breast cancer. Our results indicate that the quantitative methods convey additional information over the qualitative methods.

## Introduction

Different measures of mammographic images or density patterns [1-5] have been used as surrogate endpoints for breast cancer in etiologic research [6-9], in treatment [10-13] and in prevention trials [14-18]. Mammographic density is also important in the way in which it affects mammographic screening sensitivity [19]. It has been suggested that women with high-risk density patterns should be screened more frequently and/or with additional views per breast [20-22] or that they should be prescribed chemoprevention [23].

The first qualitative classification of mammographic density patterns was described by Wolfe in 1976 [1]. A modification of this method was proposed by Tabár [3]. Wolfe also measured the percentage of the breast containing radiographic densities on a continuous scale with the use of a polar planimeter [24]. A modification of the latter method is the BI-RADS system used in clinical radiology practice in the USA [2]. The computer-assisted technique of measuring percentage mammographic densities developed by Boyd and colleagues [4] as well as that developed at the University of Southern California [5] are other methods of quantitative assessment.

The percentage density methods and the Wolfe patterns have consistently been shown to be strongly related to breast cancer risk in different populations [6,7,15,25-29], whereas the Tabár classification has so far only been shown to be related to breast cancer risk among Chinese women [30]. Several risk factors for breast cancer have been shown to be associated with the different methods of density measurement [6-9,30-33].

The Mammography and Breast Cancer Study is a research project using mammographic density patterns as surrogate endpoints for breast cancer among postmenopausal women attending the Norwegian Breast Cancer Screening Program in Tromsø. The purpose of this study was to classify mammograms according to four methods and to examine their agreement and their relationship to selected risk factors for breast cancer.

## Materials and methods

### Study population

The study was conducted during 15 and 17 consecutive weeks in spring 2001 and 2002, respectively. In brief, women residing in the municipality of Tromsø, aged 55 to 71 years, attending the Norwegian Breast Cancer Screening Program at the University Hospital of North Norway were eligible. After giving informed consent, the study subjects were interviewed by a trained nurse about their current and previous postmenopausal hormone therapy use, reproductive and menstrual factors, previous history of cancer, and smoking status. The participants had their height measured to the nearest centimeter and weight to the nearest half kilogram. Waist and hip circumferences were measured in a standardized way to the nearest centimeter. In 2001, the women were asked to complete a four-page questionnaire at home. The questionnaire elicited information on demographic, menstrual and reproductive factors, as well as lifestyle and dietary factors. In 2002, another four pages with questions about diet were added. The National Data Inspection Board and the Regional Committee for Medical Research Ethics approved the study. Appropriate measures were taken to ensure confidentiality of the data. Altogether, 1,041 women entered the study. This accounted for 70.1% of the women attending the breast cancer screening program.

We excluded 22 women with a new or previous breast cancer diagnosis, 1 currently using chemotherapy, and 31 lacking classification from one of the three readers, leaving 987 women for analysis. Women were classified as postmenopausal if they were 56 years or older or reported having had no period during the last 12 months, or if the serum follicle-stimulating hormone level was above 20 IU/L. By these criteria three women were equivocal for menopausal status. Excluding these women did not alter the results, and they were included as postmenopausal. The readers were blinded to all characteristics of the study subjects. A total of 21 mammograms were marked as difficult to read by at least one of the readers. We reran all analyses without these mammograms, but the results were essentially the same. The 987 mammograms that were read four times were used in the analyses presented in this paper.

### Mammographic classifications

For the computer-assisted density assessments, we digitized the left cranio-caudal using both a Kodak LS-85 X-ray digitizer (reader GM) with a pixel size of 260 μm (equal to a resolution of 98 pixels per inch) and an older version of the Cobrascan scanner (Cobrascan CX 312-T) from Radiographic Digital Imaging (Torrance, CA, USA) at a resolution of 150 pixels per inch (reader GU). Reader GM used the Cumulus software from Canada [34], and reader GU applied the Madena software developed at the University of Southern California [5]. Both readers had previously successfully used these techniques for other studies [5,27,35,36]. Both computer programs assign a pixel value of 0 to the darkest (black) shade in the image and a value of 255 to the lightest (white) shade; shades of grey are assigned intermediate values. The number of pixels in each area are measured before computing the ratio between the total and the dense areas; these are then multiplied by 100 to convert them to percentages. The mammograms were classified according to reader-specific quartiles of percentage density.

The experienced radiologist (NB) [37], classifying the mammograms according to the Wolfe [38] and the Tabár [3] methods, used the latter method for the first time. In brief, the Wolfe method assigns the mammograms to four parenchymal patterns (N1, P1, P2 and DY) according to the distribution of fat and the prominence of the ducts. These patterns were again dichotomized into low-risk (N1 and P1) and high-risk (P2 and DY) patterns [38]. The Tabár method classifies the mammograms in five patterns (I to V) based on an anatomic–mammographic correlation with a three-dimensional, subgross (thick-slice) technique. Patterns 1, 2 and 3 are considered low-risk and patterns 4 and 5 high-risk. These patterns are not considered to be on a continuous risk scale. The primary difference between the Wolfe and Tabár classification systems is Tabár's pattern I; this is described in more detail elsewhere [3]. To be able to compare all three methods, we dichotomized the mammograms in the upper reader-specific quartiles to high-risk and the remaining to low-risk. We also calculated an upper quartile for percentage density based on dense area readings by reader GU and the breast area outlined for reader GM.

### Statistical analyses

#### Intra-rater and inter-rater agreement

Thirty-seven randomly selected mammograms were mixed and read blindly a second time by the three readers; the readings were 3 months apart for readers GM and NB and 18 months apart for reader GU. Subsequently, reader GU reread the dense area on a subset of 189 mammograms that had been scanned for reader GM. We calculated the percentage density from both the new and old dense area readings by reader GU.

We calculated the Pearson's correlation coefficient and the crude and weighted Kappa statistics to test the intra-reader and inter-reader reliabilities for the mammographic readings. The Kappa coefficient does not require any assumption about 'correct' categorization and includes a correction for the amount of agreement that would be expected by chance alone. A Kappa of 0% indicates that the agreement between two measurements is no greater than would be expected by chance. Kappa values of 50% or more indicate moderate agreement, 60 to 80% good agreement, those over 80% very good agreement, and those over 90% excellent agreement [39].

#### Relationship between the four methods and their associations with risk factors for breast cancer

We calculated the median percentage density for the four Wolfe and five Tabár pattern categories for each of the two readers. Similarly, we categorized the mammograms according to reader-specific quartiles with the corresponding proportion of high-risk versus low-risk Wolfe and Tabár patterns for the different quartiles. We also estimated prevalence odds ratios of having high-risk mammographic density patterns with 95% confidence intervals (CI) to express the degree of association between selected risk factors for breast cancer and the mammographic density patterns. Each of the following factors was evaluated as a potential confounder of the relation between the factor of interest and mammographic patterns: age (less than 60, 60 to 64, 65 or more), age at menarche (less than 13, 13 to 14, more than 14), age at menopause (less than 48, 48 to 50, 51 years or more), number of children (0, 1, 2, 3, 4 or more), age at first birth (less than 20, 20 to 24, 25 or more) and body mass index (BMI (defined as weight in kilograms divided by the square of the height in meters) less than 25.0, 25.0 to 29.9, 30.0 or more).

We performed multivariate analyses with models that included all the above listed variables as independent variables and the high-risk density patterns according to each of the four readings as the dependent variable. Subsequently, we reran the models with the Wolfe and Tabár patterns as outcome variables adjusting for percentage density from each of the two readers as a continuous variable. Similarly, we adjusted the models with percentage density as outcome variable for the two categories of Wolfe and Tabár patterns. Statistical trend tests were obtained by creating an ordinal exposure variable with equally spaced scores and including it in the logistic regression model. Results were considered as statistically significant if the two-sided *P *value was 0.05 or less. We performed data management and statistical analyses with the SAS statistical software package, version 8.2 (SAS Institute Inc., Cary, NC, USA) [40].

## Results

Table 1 shows the basic characteristics of the total study population and the women with mammograms in the high-risk categories. Reader GM classified mammograms with 28.3% density or more to be in the upper quartile; the corresponding figure for reader GU was 19.0% density. The value for the upper quartile was in between these, namely 21% density, when the dense area from reader GU and breast area for reader GM was used. In all, 47% and 24% of the mammograms were classified as high-risk according to the Wolfe (P2, DY) and Tabár (IV, V) classifications, respectively. Among the mammograms in the upper reader-specific quartiles, more than 95% were also classified as high-risk according to the Wolfe method, whereas the corresponding proportion for the Tabár method was less than 70% (Table 1).

The Pearson correlation coefficient was 0.93 and 0.86 for the repeated quantitative readings conducted 3 months (GM) and 18 months (GU) apart, respectively. The intra-rater agreement for the upper reader-specific quartile versus the three lower quartiles was moderate for reader GM (Kappa = 0.59; 95% CI 0.29 to 0.90) and reader GU (Kappa = 0.59; 95% CI 0.29 to 0.90). For reader NB the intra-reader agreement was good for the Wolfe classification (Kappa = 0.61; 95% CI 0.34 to 0.89) and very good for the Tabár classification (Kappa = 0.89; 95% CI 0.69 to 1.00). The Pearson correlation coefficient was 0.86 for the two original percentage density readings and 0.93 for the subset of 189 mammograms. Both the agreements between the reader-specific upper quartiles (crude Kappa = 0.69; 95% CI 0.64 to 0.74) and between all four quartiles (weighted Kappa = 0.71; 95% CI 0.68 to 0.74) were good. The agreement between high-risk and low-risk Wolfe and Tabár patterns was moderate (crude Kappa = 0.51; 95% CI 0.46 to 0.56).

Table 2 shows the distribution of mammograms according to the four Wolfe and five Tabár patterns with the corresponding median percentage density assessment for the two readers. According to reader GM the median percentage density for Wolfe high-risk patterns was 28.6% and for Tabár 34.9%. The corresponding figures for reader GU were 19.4% and 25.2%.

Table 3 shows that there was a significant inverse relation between age at screening, number of children and BMI and unfavorable density patterns according to all four classifications. Ages at menarche and menopause showed no association with any of the four outcome variables. Women who had their first child after their 25th birthday were more likely to have high-risk density patterns according to all four readings. A trend test achieved borderline significance for two of the assessments (GU (*P *= 0.07) and the Wolfe classification (*P *= 0.05)).

None of the associations displayed in the table between risk factors and the two quantitative readings changed when adjusted for the Wolfe or Tabár classification.

After adjustment for percentage density as assessed by either reader, age at entry (*P *= 0.14), number of children (*P *= 0.54) and BMI (*P *= 0.57) were no longer associated with the Wolfe classification. The association between age (*P *= 1.0) and the Tabár classification disappeared, whereas the inverse association with number of children (*P *< 0.05) and BMI (*P *< 0.03) remained statistically significant after adjustment for percentage density.

## Discussion

Our study finds that although the four methods vary depending on which mammograms are classified as unfavorable, all four methods seem to capture the same overall associations with risk factors for breast cancer. There was a high correlation for both inter-reader and intra-reader reliability between the two quantitative methods. Furthermore, mammograms classified as high-risk according to the Wolfe and Tabár classifications had the highest median percentage density. Age at screening, number of children and BMI were inversely associated with all four assessments of breast density. After adjustment for percentage density, there was no longer any association with these risk factors and the Wolfe pattern, whereas the association between number of children and BMI remained for the Tabár pattern. Controlling for the pattern classification did not change the associations between risk factors and percentage density.

All four methods of mammographic assessment have a subjective component. The percentage of dense area measurement is a ratio of two measures (dense area : total area). The mammograms in this study were rather dark, and differences in the appearance of the mammographic images due to the properties of the scanners did lead to variations in defining both areas. The two readers have both trained with Dr NF Boyd in Canada and have also worked together on previous studies [35,36]. Nevertheless, some of the discrepancy was due to differences in judgement as to what constitutes a dense area. However, the relative assessment between what constituted high-density or low-density mammograms was highly correlated. This supports the comparability of the readings.

The Kappa values for inter-rater agreement in our study are of the same magnitude as or better than those found in several other studies examining different kinds of mammographic reading. Venta and colleagues found a Kappa value of 0.46 for density measurements on X-ray and digital mammograms recorded by two radiologists [41]. In a previous study including more than 3,500 premenopausal and postmenopausal women, we found the overall agreement between high-risk and low-risk for the Wolfe and Tabár classifications to be poor (Kappa = 0.22) [3] in comparison with the moderate agreement (Kappa = 0.51) in the present study. The two classifications are not strictly independent because one reader performed both assessments. We attribute the latter higher Kappa value to the fact that all women were postmenopausal, resulting in more low-density patterns, which are easier to assess.

Our results are in agreement with a recent study describing that the Wolfe pattern classification was redundant when percentage density was available as a measure for breast cancer risk [42]. In contrast to this, the association between parity and BMI with the Tabár classification remained after adjustment for density, suggesting that this classification captures something more than just density assessments. However, we do not know whether this additional information from the Tabár classification is related to breast cancer risk. The association between age, parity, BMI and mammographic density is similar, as described in the literature [6,7]; this attests to the validity of the mammographic measurements. The results also underscore the importance of adjusting for these factors when other associations are being explored. The positive association between age at first birth and unfavorable patterns indicated in the present study has also been found in some previous reports [3,8,29,43-46], but not in others [47].

The present results are in agreement with studies finding no associations between ages at menarche [44] and menopause [47] and unfavorable density patterns, but not with others [8,45]. We found a positive association between age at menarche and unfavorable density patterns among premenopausal women and an inverse association among postmenopausal women [8]. In two other studies, a positive overall association was revealed [45,47]. In the study by El-Bastawissi and colleagues, age at menopause was positively related to unfavorable patterns [45]. In our previous study the same relationship was found to be of borderline significance [8].

Our study has several strengths. It was a part of a population-based screening project with a high attendance rate. The three readers were experienced and blinded to the risk factors. A study from the Finnish screening program using the Wolfe classification showed that at younger ages there was a greater probability of misclassification from low-risk to high-risk and at older ages there was a greater probability of misclassification from high-risk to low-risk [48]. Because the misclassification seems to be age dependent we also consider it a strength that all our women were postmenopausal. Our study is cross-sectional and does not have information on the temporal relationship between the risk factors examined and the mammographic density patterns. Several studies have shown that different types of hormone use will temporarily change mammographic density [49-51] but that there will be a reversal of the hormone-induced changes on cessation of treatment [50-52].

## Conclusion

The high-risk classification of mammograms varied to some extent according to the four assessment methods commonly used previously. Our results indicate that the quantitative methods convey additional information over the qualitative methods. Quantitative measures should therefore be preferred when high-risk density patterns are used as surrogate endpoints in etiologic research, clinical or preventive trials. The difference between Tabár and Wolfe categories in relation to breast cancer risk and quantitative density assessment needs to be investigated further. Once breast cancer screening programs start to use digital mammograms, a quantitative method may become usable also in large-scale screening programs.

## Abbreviations

BMI = body mass index; CI = confidence interval; OR = odds ratio.

## Competing interests

The authors declare that they have no competing interests.

## Authors' contributions

ITG conceived the study, had full access to all of the data in the study, and takes responsibility for the integrity of the data and the accuracy of the data analyses. Classification of mammograms was performed by NB, GM and GU. Analysis and interpretation of data were performed by ITG, YB, GU, GM and EL. ITG and YB drafted the manuscript. ITG, YB, NB, GU, GM and EL revised the manuscript critically for important intellectual content. All authors read and approved the final manuscript.

## Figures and Tables

**Table 1:** Characteristics of the study participants overall and with mammograms classified as high-risk density patterns

Characteristic	All women	Quantitative Reader GM	Reader GU/GM	Reader GU	Pattern Reader NB	
	*n *= 987	Upper quartile ≥ 28.3% (*n *= 249)	Upper quartile ≥ 21.0% (*n *= 246)	Upper quartile ≥ 19.0% (*n *= 247)	Wolfe P2, DY (*n *= 462)	Tabár IV, V (*n *= 233)
Age (years) at screening, mean ± SD	61.4 ± 4.5	60.4 ± 4.4	60.3 ± 4.5	60.3 ± 4.5	60.8 ± 4.6	60.6 ± 4.5
Age (years) at menarche, mean ± SD	13.3 ± 1.4	13.4 ± 1.3	13.4 ± 1.3	13.4 ± 1.3	13.3 ± 1.3	13.4 ± 1.3
Age (years) at menopause, mean ± SD	48.5 ± 5.1	48.5 ± 4.9	48.8 ± 4.8	48.8 ± 4.7	48.8 ± 4.9	48.7 ± 4.8
Parous (%)	92.6	89.2	88.6	87.5	89.4	88.0
Age (years) at first birth/pregnancy, mean ± SD	22.9 ± 3.7	23.9 ± 4.3	24.1 ± 4.3	24.1 ± 4.3	23.4 ± 3.9	23.9 ± 4.4
Number of children, mean ± SD	2.6 ± 1.4	2.1 ± 1.2	2.1 ± 1.1	2.0 ± 1.2	2.3 ± 1.3	2.1 ± 1.2
Body mass index, mean ± SD	27.4 ± 4.8	24.9 ± 3.6	25.1 ± 3.7	25.1 ± 3.8	26.0 ± 4.0	25.9 ± 3.8
Wolfe high-risk patterns (P2, DY) (%)	46.8	95.6	96.3	96.4	-	99.1
Tabár high-risk patterns (IV, V) (%)	23.6	60.6	66.3	67.2	50	-
Reader GM						
Median density, % (range)	14.5 (0–88.4)	-	41.1 (9.4–88.4)	39.4 (7.6–88.4)	28.6 (1.9–88.4)	34.9 (1.9–88.4)
Reader GU						
Median density, % (range)	9.6 (0–69.2)	26.6 (0.6–69.2)	27.7 (0.6–69.2)	-	19.4 (0.6–69.2)	25.2 (3.5–69.2)
Readers GU and GM						
Median density, % (range)		29.5 (3.9–73.8)	-	30.5 (18.4–73.8)	21.7 (2.1–73.8)	28.1 (3.6–73.8)

For percentage mammographic densities, the upper quartile is defined as high-risk and the lower three quartiles as low-risk.

**Table 2:** Agreement between two qualitative (Wolfe, Tabár) and two quantitative (median percentage density measurements) classifications

	Totals	Wolfe				
			
		N1	P1	P2	DY	
Tabár		190, *2.6*, **0.4**	335, *9.4*, **5.5**	228, *21.5*, **15.5**	234, *39.5*, **26.5**	
I	470, *15.0*, **9.4**	0	255, *9.2*, **5.1**	165, *22.1*, **13.9**	50, *39.3*, **19.8**	Tabár low-risk: 754, *10.1*, **6.0**
II	194, *2.7*, **0.4**	188, *2.6*, **0.4**	6, *8.6*, **2.9**	0	0	
III	90, *12.3*, **8.6**	1, *4.8*, **1.8**	73, *9.9*, **7.6**	14, *19.5*, **15.8**	2, *34.0*, **14.6**	
IV	159, *30.3*, **23.7**	1, *41.8*, **22.1**	1, *9.2*, **14.4**	49, *20.5*, **18.8**	108, *36.4*, **27.3**	Tabár high-risk: 233, *34.9*, **25.2**
V	74, *46.4*, **28.1**	0	0	0	74, *46.4*, **28.1**	
		Wolfe low-risk: 525, *6.3*, **2.8**		Wolfe high-risk: 462, *28.6*, **19.4**		

Measurements by reader GM (with Cumulus software) are shown in italics, and by reader GU (with Madena software) in bold. Total *n *= 987 women.

**Table 3:** Associations between selected risk factors for breast cancer andmammograms classified according to four methods

Characteristic	Upper quartile		High-risk patterns Reader NB	
			
	Reader GM ≥ 28.3%	Reader GU ≥ 19.0%	Wolfe	Tabár
Age at screening				
<60 years	1.00	1.00	1.00	1.00
60–64 years	0.65	0.74	0.64	0.79
65+ years	0.52	0.53	0.59	0.64
*P *for trend	<0.01	<0.01	<0.01	<0.03
Age at menarche				
<13 years	1.00	1.00	1.00	1.00
13–14 years	1.24	1.28	1.26	1.36
14+ years	0.90	1.04	0.81	0.76
*P *for trend	0.99	0.50	0.67	0.70
Age at menopause				
<48 years	1.00	1.00	1.00	1.00
48–50 years	0.80	0.85	0.90	0.87
51+ years	0.95	1.14	1.38	1.11
*P *for trend	0.83	0.47	0.06	0.61
Age at first birth				
<20 years	1.00	1.00	1.00	1.00
20–24 years	0.99	1.15	1.41	1.14
25+ years	1.30	1.66	1.67	1.40
*P *for trend	0.27	0.07	0.05	0.15
Number of children				
0	1.00	1.00	1.00	1.00
1	1.14	1.54	1.61	1.16
2	0.52	0.87	0.87	0.40
3	0.51	0.60	0.90	0.37
4+	0.18	0.23	0.41	0.21
*P *for trend – all	<0.0001	<0.0001	<0.0001	<0.0001
Body mass index				
<25	1.00	1.00	1.00	1.00
25–30	0.39	0.48	0.53	0.81
>30	0.15	0.19	0.24	0.36
*P *for trend	<0.0001	<0.0001	<0.0001	<0.0001

Multivariate odds ratios have been adjusted for age at screening, age at menarche, age at menopause, number of children, age at first birth, and body mass index where applicable. Total numbers in the multivariate models may vary slightly owing to missing values for covariates.
